# No one-size-fits-all: Multi-actor perspectives on public participation and digital participatory platforms

**DOI:** 10.1098/rsta.2024.0111

**Published:** 2024-11-13

**Authors:** J. E. Gonçalves, I. Ioannou, T. Verma

**Affiliations:** ^1^ Department of Urbanism, Technical University of Delft, Julianalaan, Delft 134 2628 BL, The Netherlands; ^2^ Department of Multi-Actor Systems, Technical University of Delft, Jaffalaan 5, Delft 2628 BX, The Netherlands

**Keywords:** public participation, citizen engagement, digital technologies, urban platforms

## Abstract

This paper explores the perspectives of different urban actors regarding public participation in the context of the increasing incorporation of digital technologies and urban platforms. The study is based on three workshops with local governance actors, six semi-structured interviews with academics in the fields of public participation and digital technologies and a citizen survey with 260 respondents. The results provide multi-perspective insights into the challenges of participatory processes and are synthesized into three contributions: (i) guidelines for effective public participation, including factors that encourage or discourage citizen engagement; (ii) guidelines for designing participatory platforms, highlighting specific features that promote digital engagement (i.e. social media, gamification and user-friendly interfaces), and (iii) a typology of digital participation platforms to connect the diverse needs of actor groups with the various possibilities provided by new technologies. The guidelines provide concrete recommendations to support both urban practitioners and interface designers in designing participatory strategies and platforms, respectively. Recognizing that there is no-one-size-fits-all platform, the typology provides a framework for the assessment and further development of digital platforms for public participation.

This article is part of the theme issue ‘Co-creating the future: participatory cities and digital governance’.

## 1. Introduction

Cities are complex systems, an intricate mixture of nodes, networks, places and flows, where various relations, activities and values coexist and interact [1,2]. Within these complex environments, various interest groups collaborate and compete to achieve their objectives. Urban decision-makers thus require varied methodologies and tools to capture the diverse needs and aspirations of these groups and address the practical challenges and value conflicts that emerge from this complexity [3]. An important shift in this direction occurred with the introduction of participatory practices in urban planning to capture contextual dynamics and local knowledge and ensure the legitimacy of planning decisions [4,5]. Since then, public participation has become an integral part of planning and policy-making processes [6,7]. There are different participation formats from conventional participation formats, such as public hearings and meetings; other forms of engagement range from listening and learning sessions, competitions, interactive websites and working groups [8] to more radical forms of radical democracy, such as participatory budgeting, liquid democracy, citizen assemblies and mini-publics [9–12]. However, the emergence of participation in urban governance is not without criticism. Factors such as power imbalances, tokenistic engagement and limited representation often undermine the potential of participatory practices, raising questions about their real impact on decision-making processes and outcomes [13–16]. Others add that the institutionalization of participation has reduced it to a series of mandatory methodological packages in the form of checkboxes with no transformative, empowering or democratic purpose [15,16]. Moreover, public institutions are increasingly outsourcing participation to private consulting companies—who have a responsibility to their shareholders and not to the public good—further eroding public trust and preventing in-house capacity-building [16,17].

In addition to the inherent complexities of urban governance, the increasing digitalization of public participation introduces its own set of opportunities and challenges. Digital technologies have the potential to streamline stakeholder involvement, overcoming constraints of time, space and participation capacity associated with traditional methods [6,18–23]. Scholarly discourse also underscores the role of technology in facilitating civic engagement in collective decision-making, fostering transparency and accountability in governance and democratizing decision-making processes [13,24–28]. While digital technologies have the potential to enhance accessibility, engagement and inclusivity, they also bring forth a range of issues that can influence the effectiveness and trustworthiness of participatory processes. Issues such as sample size, sampling methods, bias and data accuracy affect the quantity and quality of the information gathered through digital technologies [18,29]. The digital divide, training needs, costs and socio-political context further challenge the implementation and uptake of digital participation technologies [13,30–33]. In response, the literature highlights the need to establish ethical guidelines and value-sensitive principles for digital governance [28,34], further reinforced by calls for technological sovereignty based on alternative practices and infrastructures of open-source digital development, ethical procurement practices, and digital democracy, among other actions [35–37].

Various academic frameworks have been developed to unpack the complexity inherent in public participation, offering conceptual insights to understand the dynamics of stakeholder engagement, power relations and decision-making structures. One of the first scholars to conceptualize public participation was Sherry Arnstein. Amidst civil rights movements, anti-war protests and countercultural movements in the late 1960s, Arnstein conceptualized public participation as levels of power distribution through which the ‘have-not’ citizens and communities can be deliberately included in decision-making processes [38]. Through the same critical lenses, Pretty [39] derived a typology of participation that considers how (material) resources interact with power dynamics [39]. By analysing the interests of both facilitators and participants within participatory processes, White [40] identified four major forms of public participation: nominal, instrumental, representative and transformative [40]. Two other important typologies are three-dimensional: The Democracy Cube [41] represents public participation using three dimensions, including authority and power levels, intensity of communicative exchange and degree of participants' inclusivity, while the PowerCube Framework [42] explores power dynamics within participation processes across the three axes of levels, spaces and forms of power. A recent contribution to the conceptualization of public participation is the 3A^3^ framework [8], a multi-dimensional framework that embeds participation in planning processes and the wider context.

While these frameworks help to make sense of the complexities involved in public participation, their application to assess the effectiveness of participatory practices is often challenged by the reasons that prompt stakeholders to engage in public participation. For instance, processes that empower participants in decision-making would be deemed effective through the lenses of Arnstein and Pretty. By contrast, some scholars argue that citizen empowerment may not always be desirable, especially when decisions impact a broader community beyond participants [43], and that contemporary challenges, such as climate change, depend on new forms of social learning that contest not only the means but also the meaning of participation in policy-making processes [44,45]. In literature, these debates are often encapsulated in a binary discussion, in which public participation is approached either from a normative/critical or a pragmatic/optimistic viewpoint [46,47]. A normative perspective argues that including the public in decision-making is essential to ensure legitimate planning outcomes [13,41]. By contrast, a pragmatic approach to participation contends that by tapping into public/local knowledge and expertise, public participation leads to more innovative and context-appropriate solutions [13,48]. In practice, participatory processes may be driven by both normative and pragmatic reasons, further complicating the question of participation effectiveness.

Going beyond debates of whether public participation improves planning and policy outcomes or whether it delivers on its promises of straightening democratic values [14,49,50]—or both or neither—it is important to understand how urban actors perceive the complexity of participatory processes and the meaning of effective participation [51,52]. In most cases, participation is evaluated in the absence of the participants on the basis of criteria derived from theory and/or the analysis of cases [46]. The few scholars who have examined stakeholders' perceptions of public participation have often done so within a specific case, such as infrastructure projects or environmental issues, e.g. [9,53–56]. While these studies are essential to address issues of participation in concrete cases, their case-specificity leaves less room to understand emerging trends, patterns and issues in participation. A smaller portion of the literature focuses on perceptions of public participation across different cases or detached from specific cases (but not from specific contexts), and those have often focused on gathering input from public administrators and managers, private actors and researchers [18,57–60]. To the best of our knowledge, a broader approach to public participation perception that includes citizens and other local perspectives is still missing.

This study aims to uncover the perspectives of urban actors on the following topics: (i) the complexity of public participation in urban governance, (ii) the meaning of effective public participation, and (iii) the use of digital platforms for public participation. The study is based on three workshops with local governance actors,^1^ six semi-structured interviews with academics in the fields of public participation and urban digital technologies, and a citizen survey with 260 respondents. The results provide multi-perspective insights into the challenges of participatory processes and are synthesized into three contributions: (i) guidelines for effective public participation, including factors that encourage or discourage citizen engagement, (ii) guidelines for designing participatory platforms, highlighting specific features that promote digital engagement (i.e. social media, gamification and user-friendly interfaces), and (iii) a typology of digital participation platforms to connect the diverse needs of actor groups with the various possibilities provided by new technologies. By considering the perspectives of different urban actor groups, this research seeks to translate these insights into concrete contributions to support public participation, particularly in the context of increasing incorporation of digital technologies in urban governance.

## 2. Methodology

**Figure 1.**
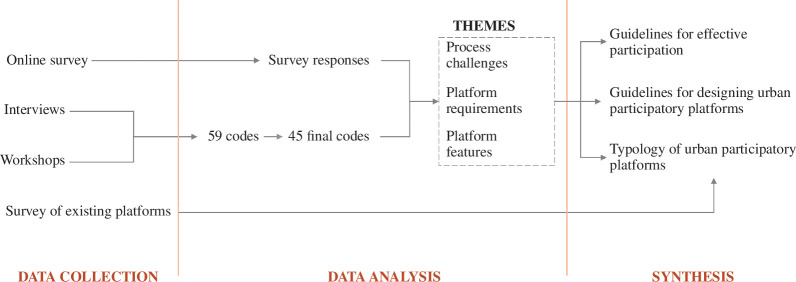
Overview of the research approach in three phases: data collection, data analysis and synthesis.

The research approach in this research comprises three phases: data collection, data analysis and synthesis. The data collection was structured around the three topics previously mentioned: (i) the complexity of public participation in urban governance, (ii) the meaning of effective public participation, and (iii) the use of digital platforms for public participation. Three actor groups were targeted in this study: local governance, academia and citizens. The choice of actors aims to complement the existing literature, which often focuses on one group at a time, usually public administrators, private actors or researchers. Different methods were used to approach three urban actor groups. Next, the data analysis phase involved coding the qualitative data and analysing the survey statistics. Three themes were derived from the codes, namely process challenges, platform requirements and platform features. The results were then synthesized into guidelines for effective participation, guidelines for designing digital participation platforms and a typology for digital participation platforms. Finally, a survey of existing platforms was conducted to corroborate the pertinence of the platform typology. An overview of the research approach is shown in figure 1.

### (a) Data collection and context

Given the multi-actor nature of this study, different data collection methods were used to approach each urban actor group. Workshops targeted practitioners and policymakers, semi-instructed interviews targeted academics and researchers and an online survey targeted individual citizens. The results from the first workshop, in combination with the literature, were used to develop the semi-structured interview guide. Following the same approach, the results from interviews and workshops were used to develop the questions for the citizen survey. By doing so, expert input (from practice and academia) collected via interviews and workshops could be contrasted with citizen input on the same topics. Here, it is important to note that the study started with a rather narrow definition of what a digital platform is: ‘a specific type of civic technology explicitly built for participatory, engagement and collaboration purposes that allow for user-generated content and include a range of functionalities (e.g., analytics, map-based and geo-located input, importing and exporting of data, ranking of ideas) which transcend and considerably differ from social media such as Social Networking Sites and Microblogging (such as Facebook, Twitter and Instagram)’ [62]. The results from workshops and interviews challenged this definition and prompted us to add questions about the use of social media and other websites for engagement in the citizen survey. Moreover, we highlight that our study is largely bound to the Dutch context: local governance actors include local government employees, applied researchers, and practitioners working in/with Dutch local municipalities; academic actors cover researchers focusing on both Dutch and international contexts and citizen insights were collected worldwide but are biased to the Dutch and Greek contexts. The implications of this contextual bias are discussed later in the paper. The following sub-sections provide more details on each data collection method.

#### (i) Workshops

In total, three workshops were conducted, focusing on practice input. Participants were purposely selected to provide ‘insider’ knowledge of the local governance practices related to participation and digitalization/data management (electronic supplementary material, table A1). The sessions were organized over the course of eight weeks from March to May 2022, either online or in a physical setting. In the first workshop, we conducted a stakeholder mapping exercise to identify users of public participation platforms, what information they may expect from each other and the factors that influence their participation. In the second workshop, we interacted with data experts working in public institutions. Here, we gathered information about existing participatory platforms, issues related to data privacy and security and their practical experience with maintaining public digital platforms. In the third workshop, we discussed our preliminary findings with applied researchers and practitioners, particularly the requirements and features for digital platforms and the typology of public participation platforms. The data were gathered on an online collaborative whiteboard platform in the first workshop and paper canvases and thick notes in the other two workshops.

#### (ii) Interviews

Six semi-structured interviews were conducted, targeting academic input. Interviewees were purposely selected to represent the different knowledge fields relevant to the three topics emerging from the literature: (i) the complexity of public participation in urban governance, (ii) the meaning of effective public participation, and (iii) the use of digital platforms for public participation. The interview invitations were sent via email (21 invitations in total, with six positive replies). The final interviewee list included experts related to urban planning, data science and digital technologies (electronic supplementary material, table A2). The interviews were conducted online, in English, via Microsoft Teams in May 2022. An interview guide was developed based on the three topics described above (electronic supplementary material, table A3). The interview guide was a living document during the interview round, and the questions and/or the focus of the interview were adapted to the interviewee’s profile. After the completion of every interview, the transcription was automatically generated by Microsoft Teams. The transcribed text was afterwards carefully checked for potential inconsistencies to secure the maximum level of a literal transcription. Interruptions during the interviews were signalled and not analysed.

#### (iii) Citizen survey

The survey targeted citizen input, aiming to cover the same topics as the workshops and the interviews. However, the questions were adapted (i) to the audience by removing technical terms and (ii) to the data collection method by replacing open questions with closed or short-answer questions. The intention was to keep the survey relatively short. The survey questions are presented in the electronic supplementary material, table A4. The survey was created and further analysed using Qualtrics software. The survey was distributed in various ways, including the circulation of a link via email to potential respondents and a QR code that was published and promoted on the researchers’ social media network. The survey was also promoted offline at the Delft University of Technology and in local establishments in the city of Delft, The Netherlands, aiming to target older people with limited or no access to social media. The survey was open for four weeks during May and June 2022, and 260 answers were collected. The demographic profiles of respondents are presented in the electronic supplementary material. Despite our efforts to distribute the survey and broaden the spectrum of respondents, most answers came from large cities in The Netherlands and Greece, which prevents any statistical inference that generalizes the insights provided here to a larger population. Instead, we approach the survey data from an exploratory point of view, aiming to verify the trends, patterns and areas of interest identified in the workshops and interviews without necessarily drawing broader inferences. Here, it is important to highlight that the respondent sample covered citizens with and without previous experience with digital tools for public participation (53% and 47%, respectively), potentially giving us a balanced view of digital participation.

#### (iv) Survey of existing platforms

A survey of existing platforms was conducted to inform the typology development. More than 150 platforms have been identified, including the 113 platforms identified by Falco & Kleinhans [62] and an online list by the International Society for Participatory Mapping. Additional platforms were identified through desk research and suggested by the workshop participants. The final list of platforms is presented in the electronic supplementary material, table A5. The platforms have not been systematically analysed in this work, but a few of them are cited as illustrative examples for the proposed typology.

### (b) Data analysis

#### (i) Workshops and interviews

The analysis of both workshop and interview data was conducted using the online software ATLAS.ti. Two types of coding were used for the data analysis in this research: focus and open coding [63]. The focus codes were based on three topics of tension emerging from the literature: (i) the complexity of public participation in urban governance, (ii) the meaning of effective public participation, and (iii) the use of digital platforms for public participation. Open codes were helpful in capturing the level of detail in the data, which was challenging with the focus codes. The analysis led to the creation of 251 quotations. These quotations were labelled with 59 different codes, which were reduced to 45 codes after refinement (i.e. merging similar codes or deleting repeated ones). After realizing that the focus codes did not provide a coherent basis to describe the data collected, the 45 codes were clustered into three themes: process challenges, platform requirements and platform features.

#### (ii) Citizen survey

The survey responses were analysed in two stages using qualitative and quantitative methods. The data were summarized and aggregated using the Qualtrics software. The survey was qualitatively analysed based on the codes from the analysis of the interviews and the workshops. The processes of summarization and aggregation also helped identify the patterns in the data using statistics (aggregations of frequency distribution and cross-tabulation technique). In this paper, we present the survey results as a group; however, the cross-tabulation analysis provided some insights about differences across gender, age and income and is presented in the electronic supplementary material, part B.

### (c) Synthesis

In the last research phase, the results were synthesized into three concrete contributions: (i) guidelines for effective participation, (ii) guidelines for designing digital participation platforms, and (iii) a typology for digital participation platforms. The guidelines were synthesized from the workshop and interview codes and the survey data in two steps: cross-comparison between the three groups to identify trends and tensions and integration to deliver guidelines that address the needs of all actors. This means creating both ‘shared’ guidelines and guidelines that address the needs or concerns of a specific group. The platform typology emerged from existing conceptualizations of public participation [38–40], the multi-actor insights of this research and the survey of existing platforms. By triangulating these sources, we delineated five characteristics that help differentiate urban platforms, from which we identified six types of platforms with concrete examples of each.

## 3. Results

The results are presented in three sections. First, the three themes resulting from the qualitative coding are described. Theme 1: process challenges refers to perspectives related to challenges of public participation within urban governance; theme 2: platform requirements refers to perspectives on the must-haves of digital participation platforms, encompassing aspects of, e.g. language and privacy; and theme 3: platform features includes platform design elements that address challenges and/or achieve platform requirements.

### (a) Workshops

#### (i) Process challenges

Most workshop participants highlighted the complexity of managing the various and diverse stakeholders involved in the planning process as a critical challenge. Clear communication and transparency among stakeholders were identified as crucial aspects of dealing with this challenge. Neighbourhood managers and public officials working at the ‘street’ level considered it important to be informed of policies and monitor how their neighbourhood ‘is doing’ compared with other neighbourhoods in the city. They also emphasized the necessity for a designated moderator to clarify the positions and responsibilities of urban stakeholders, outline expected outcomes and ensure an open and transparent relationship among them. Contextuality also emerged as a key challenge in public participation, as stakeholder engagement heavily relies on the specific context. Participants related the challenge of contextuality to the challenge of inclusiveness, as ensuring an inclusive approach to participation requires the development of a flexible ‘toolbox’ of participation methods that can adapt to diverse contexts. The discussions touched briefly on the topic of using rewards to incentivize stakeholder engagement, noting that different stakeholders require varied incentives to encourage their participation.

Furthermore, participants emphasized the importance of enhancing collaborations between citizens and other urban stakeholders to enrich the public participation process. Finding effective ways to collaborate with citizens, foster mutual trust and work together was seen as imperative for successful engagement in urban planning: ‘We need to find an effective way to collaborate with citizens and work together and build mutual trust’ (participant, workshop 2). Several participants highlighted the potential of digital platforms in fostering dialogue among urban stakeholders to establish deeper connections and build a shared knowledge base. They also see potential in using digital platforms to foster a sense of community: strengthening social networks and local support structures, facilitating mutual connections and supporting neighbourhood initiatives were underscored as means to empower communities. However, they note that building a strong community requires a gradual, step-by-step approach. Furthermore, insights into the use of digital platforms include the importance of ensuring inclusivity and the necessity of tailoring the platform to both the audience and the context.

Workshop participants placed a significant emphasis on citizens and communities. One participant from workshop 2 highlighted the municipality’s interest in fostering citizen engagement: ‘The municipality is interested to see how people reflect. We want to give people more say in their neighbourhoods. Build a link between citizens and policymakers.’ They emphasized the value of local citizens' perspectives in identifying both strengths and weaknesses within neighbourhoods to inform discussions and the co-creation of new policies and tools. However, it was noted that understanding the motivations and preferred methods of citizen participation is crucial. A participant from workshop 3 emphasized the importance of creating an engaging experience beyond mere surveys, stating, ‘If it is just a survey, people will skip it. It is about the whole experience.’

Based on their previous experience with public participation, the workshop participants were able to provide insights into barriers related to citizen engagement, including the reluctance of citizens to report problems due to fear of stigmatization, scepticism about whether their input will be valued by decision-makers and practical barriers such as language and time constraints. Additionally, insights were provided into the challenges citizens face when using digital participation platforms, such as difficulty expressing thoughts or lacking background knowledge on specific topics. To address these barriers, it is essential to collect citizen input thoughtfully, ensuring relevance to daily concerns and facilitating meaningful participation: ‘It is important to find the right questions and the way that will help to get fruitful answers’ (participant, workshop 3).

#### (ii) Platform requirements

During discussions about platform requirements, workshop participants highlighted the need for a modular and adaptive platform design, emphasizing the importance of platform flexibility to accommodate diverse use cases and user preferences, depending on the context. Most participants emphasized the need for easy-to-use platforms and user-friendly designs. The use of visually appealing and engaging content, along with a fun-to-use interface, also emerged as factors that can enhance user engagement with the platform. They also highlighted the importance of intuitive layouts for ensuring inclusivity. By contrast, language features, including formal versus informal language use and terminology choices, were emphasized for potential exclusionary effects. As a participant from workshop 2 emphasized: ‘Be aware of the feeling of people when they know that their neighbourhood has problems. There is a need to specify the problem, but people get stigmatised if you do not use the right words.’

Another interesting requirement that emerged from the workshops was the need to ensure that users feel confident in collaborating with one another on digital platforms. Knowing that platform users and collaborators are trustworthy is, therefore, key for stakeholders to be willing to cooperate with each other in digital environments. Workshop participants also mentioned requirements related to data. First, there was an emphasis on integrating both quantitative and qualitative data. A participant from workshop 1 articulated the importance of gathering subjective information such as personal experiences, stories and local history, while another participant from workshop 2 supported this perspective by suggesting that combining qualitative data with municipal data (such as census data, statistics, surveys and other (quantitative) sources) could enhance public participation in digital platforms. Second, participants stressed the importance of ensuring the privacy and security of personal data. This discussion also covered the challenge of finding the balance between data openness and security. Finally, the frequency of data updates was deliberated upon, revealing that while regular data updates can be beneficial, their necessity varies depending on the data type. For example, census data are updated every 10 years, while policies and programmes are monitored on a yearly basis. In addition, as expressed by a participant from workshop 2, ‘There is a trade-off between impact and effort in order to decide on the update of data’.

#### (iii) Platform features

In relation to the platform features, the input gathered from the workshops was not extensive, as the discussion did not delve deeply into this aspect. However, as interviews were conducted concurrently and some platform features were already identified, participants in workshop 3 were explicitly asked about platform features. Here, gamification and mapping functionalities were highlighted for their potential to enhance engagement and contextual understanding and facilitate knowledge exchange among stakeholders. Other features included the use of QR codes across the city to bring citizens from the physical to the digital environment, as well as storytelling methods to connect planning issues to the daily concerns of citizens. Finally, participants highlighted the importance of tailoring these features to stakeholders/platform users.

### (b) Interviews

#### (i) Process challenges

Interviewees reiterated the complexity of planning and policy processes and the management of stakeholder relations as fundamental issues in public participation. Stakeholder expectation management was identified as another challenge: ‘When you do some sort of participatory process, you would need to set the expectations clear so that it’s not people don't come in there with expectations that some things would happen. And then, in the end, they will be even more frustrated than they were before’ (interviewee 1). When asked about the effectiveness of public participation, most interviewees mentioned that effectiveness is a vague term. They also noted that there is a distinction between achieving effective outcomes and implementing effective processes, highlighting the need to consider both aspects for successful participatory planning initiatives. Nevertheless, they provided various indicators associated with effectiveness, including sustainability, continuity, transparency, reciprocity, equity and heterogeneity, highlighting the multi-dimensional nature of effectiveness. One interviewee stressed the significance of continuity and long-term engagement, advocating for a thorough approach from the beginning, including identifying stakeholders, timing engagement and maintaining ongoing dialogue until the conclusion of the process and beyond. Particularly concerning citizen engagement, interviewee 4 noted that ensuring citizens' input is deeply valued and meaningfully incorporated throughout the entire process is essential for effectiveness, while another interviewee noted the importance of long-term engagement for effective citizen participation.

Interviewees recognized the importance of citizen perspectives in planning decisions while stressing that inclusivity is necessary and that ‘everybody is being included in this process and not the ones that have like the biggest or the loudest voice’ (interviewee 3). They highlighted that some groups are easier to engage because they are well organized, while some individuals and communities are significantly harder to reach, especially as they may represent voices that are seldom heard or often overlooked. Moreover, while inclusion was considered essential, interviewees also pointed out that it is equally important to assess whether individuals feel included, as there can be a disconnect between being included in the process and feeling genuinely valued and heard. Moreover, they reveal that there is resistance from policymakers to involve citizens in urban planning and policy due to the dominant top-down approach in urban governance. Interviewees also expressed concerns about political considerations and institutional distrust. Political affiliations and the credibility of those leading the projects influence individuals' willingness to engage. They cited past negative experiences with participatory initiatives and questioned the intentions of municipal involvement. Other prominent challenges include a lack of interest in participating, differing priorities of stakeholders and a lack of knowledge base across stakeholders.

Concerning factors that influence citizen engagement, many interviewees highlighted the use of rewards as a key factor to ensure participation. Whether monetary or not, rewards serve not only as incentives for participation but also ‘to give something back’ to people, taking forms such as financial compensation, gifts, discount codes or certificates. While some mentioned that financial compensation could be not only effective but also fair, as people invest their time and provide their input, others cautioned against overusing rewards to avoid influencing or manipulating citizen opinions: ‘But rewards also must be carefully used because you don't want to use the reward as bait to influence or manipulate the opinion of the citizen’ (interviewee 2). One interviewee also noted that people may not expect compensation but can still be motivated by intrinsic factors, such as a sense of ownership, solidarity and community building, awareness of social issues and contributing to democracy. Furthermore, interviewees stress that people are more likely to participate if they believe their opinions can make a difference. The sense of self-efficacy, where individuals perceive their contributions as impactful or sparking meaningful discussions, was emphasized by interviewee 1. Here, the importance of connecting to the everyday concerns of citizens was also seen as a motivating factor. Personal development and ‘selfish’ motivations were also noted as potential encouragements.

Regarding the use of digital platforms in public participation, interviewees suggested a hybrid version of participation, including both digital and in-person participatory activities, while noting that combining different methods is challenging. Aspects to consider are scalability, time and (institutional) resource issues, as well as the reach and the level of participation that can be achieved. In addition, they underline that public participation tools and methods are context-dependent and need to be tailored to the audience with a range of options suitable for everyone. In the case of digital tools, they highlighted several factors that may discourage engagement, including digital literacy, time constraints, resource limitations (i.e. not owning a digital device) and language barriers (for non-native speakers). One interviewee noted the importance of feeling comfortable with digital tools, particularly in tasks like marking points on a map, which may require a certain level of familiarity with computers, tablets or smartphones. Not being able to interact with digital technologies may lead to feelings of frustration, hindering engagement.

#### (ii) Platform requirements

Interviewees saw the potential of digital platforms as a tool to create dialogue and build a knowledge base between stakeholders, keeping relevant stakeholders well informed through a transparent process. Interviewees stressed the importance of a user-centric design approach, as clearly indicated by interviewee 2: ‘Don't design [a platform] and then think of the audience. […] I think is a golden rule for anything you want people to use.’ In addition, appealing and user-friendly interfaces were considered as factors to attract more users to the platform. In addition to being an engagement factor, it was underlined that user-friendliness could affect the quality of the input collected by the platform: ‘But if users don't understand how to use the platform, then that also interferes with your data quality’ (interviewee 4). Interviewees reiterated the importance of language usage and identified the style of the platform as a crucial factor, stressing the need for simplicity and clarity in the interface design to minimize cognitive overload. However, trade-offs between simplicity and user experience were also highlighted. One participant succinctly expressed this by stating, ‘Basic survey works as effectively as a sophisticated mapping survey’ (interviewee 2). Conversely, another interviewee noted, ‘I think that map-based surveys are fun to use compared to a normal survey’ (interviewee 4).

The interviewees reiterated the importance of understanding the context and the users. Interviewee 3 highlights that a tailor-made way of participation is needed to address different groups according to their skills, time and interests. The idea of a platform that works as a ‘toolbox of participation’ emerged from this discussion, underscored by requirements such as modular design and adaptive features. They emphasized that the platform’s overall design should be modular to ensure flexibility and adaptability to change. By incorporating different modules and features that can be added or removed, the platform can be customized to provide tailor-made solutions for specific needs and users. Here, interviewees also highlighted the importance of having an open-source platform (in the sense of open software). An open platform helps to ensure transparency and can serve as a basis for others to build upon, modifying features and including new ones, contributing to both open governance and open science.

The interviews also revealed trade-offs between transparency and privacy. An important requirement that many interviewees underlined was the need to make data open for platform users to ensure transparency. Interviewees emphasize that citizens want to have access to the platform contributions and the general results of the process so they can understand ‘who says what’. The verification of platform users and the clear definition of relationships among them can increase the transparency of the process, as well as its trustworthiness. Here, the involvement of municipalities in participatory activities, including the ownership of digital platforms, was cited as an important factor for credibility. In addition, adding user verification to the platform was also mentioned as a way to prevent bots and people with multiple accounts. However, concerns were raised regarding citizens' reluctance to share personal data, which could deter their participation. Moreover, interviewees pointed out the potential barriers posed by these privacy requirements, including bureaucratic processes and time-consuming procedures. Interviewee 3 suggested a solution involving anonymous login methods to verify users' identities while keeping their information anonymous. Interviewee 5 suggested reconsidering the necessity of stringent privacy measures for non-personal questions that provide valuable information without ethical concerns. Finally, a moderator managing the platform was also underlined as a critical requirement. Here, the moderator takes the role of making sure that interactions on the platform are safe and respectful.

Since the interviews targeted academic input, the interviewees were specifically questioned about the quality and quantity of the input people can provide through a digital platform. Most interviewees underlined that input quality and quantity are equally important. Additionally, they stated that the two terms, in several cases, are interrelated and encapsulated in each other: ‘I think the quality is most important. But for me, quality also means quantity. That all perspectives have been included’ (interviewee 3). Another interviewee stresses that quantity is not quality: ‘The most important is to make sure that all participant groups that are affected by this are included in terms of age, gender and socioeconomic status. […] If you have a large quantity, but it’s only men of a certain age group. That’s not participation’ (interviewee 2). In addition, interviewees reiterated the potential of qualitative data to enrich existing quantitative datasets (such as census and other surveys). Interviewees acknowledge that the discussion on the quality and quantity of people’s input is context-dependent; they both depend on the type of project, the tools in use and the knowledge required at the different stages of the planning process.

#### (iii) Platform features

During the interviews, mapping features emerged as a pivotal feature in digital platforms for urban planning and policy. Maps were appraised for providing a tangible medium to facilitate exchange among stakeholders, as the possibility of locating specific aspects or topics on a map offers both an overall impression of the city (zoom-out) as well as a localized understanding of specific areas (zoom-in). Interviewees emphasized the value of using maps to present various scenarios for the city, making different perspectives more concrete. In addition, according to interviewee 2, using maps is particularly interesting for citizen engagement, as they bring complex topics closer to the citizens’ everyday lives, enabling them to provide more informed input and enter discussions with enhanced knowledge. Mapping features were also seen to enhance the collection of temporal and behavioural data and provide insights into how people move within the city and what patterns emerge from this movement. However, potential hindrances were also identified. Some interviewees noted limitations with citizen engagement on large-scale maps, making it challenging for citizens to provide meaningful information. Suggestions were made to incorporate landmarks to assist orientation and to ensure map accessibility for individuals with disabilities, such as colour blindness and dyslexia.

Gamification and social media features were suggested as methods to capture attention, although considerations regarding age and accessibility were noted. According to interviewee 3, gamification could enhance the process in various ways, including making participation more enjoyable and fun for users while also fostering understanding through roleplay-based games. In the latter case, by stepping into the role of others, stakeholders could better comprehend different perspectives, including governmental limitations, thus improving their understanding of decision-making processes. In addition, interviewee 4 highlighted the potential of gamification to offer intangible rewards that could further enhance and sustain engagement: ‘A game about a neighbourhood and people were playing in their neighbourhood as avatars and creating different spatial scenarios for their neighbourhood. You could have something like a reward within the game itself.’ However, concerns were raised regarding the potential exclusion of certain demographics, such as older individuals or those unfamiliar with gaming experiences. With the incorporation of social media features, interviewee 3 sees the potential of digital platforms to serve not only as a means for public participation but also as a way for individuals to connect socially, particularly within their neighbourhoods. In addition, interviewee 4 emphasized the importance of social media features for advertising and reaching a wide audience: ‘Social media can be quite effective in terms of really having targeted outreach to communities’.

Other features highlighted by the interviewees included audio recordings, wearable technologies, three-dimensional models and visualizations and Light Detection and Ranging (LIDAR) scanners. Audio recordings were also mentioned as a feature that can be used for the platform. The convenience and briefness of collecting speech messages were underlined. However, several interviewees posed issues with data privacy, as well as problems related to automated text analysis. Wearable technologies, such as smartwatches, were also proposed as a way of behavioural and mobility data collection, but similar concerns about data privacy were raised. LIDAR scanners were proposed as a novel method for citizen-driven data collection. Interviewee 1 noted that, with the integration of LIDAR scanners in mobile devices, users could conduct three-dimensional scans of locations, thereby enriching the data collection process with detailed spatial information. By contrast, three-dimensional models and visualizations were suggested as a feature to enable users to comprehend the urban environment more easily. Interviewee 1 provided an example where users could explore different park designs by placing three-dimensional models of various flowers and plants from different parts of the city, enhancing the understanding of proposed changes in an immersive experience.

Finally, interviewees emphasized the importance of the platform’s modularity with respect to features. This entails features being adaptable across various circumstances and user needs. It was noted that features should cater to different types of users, such as researchers, policymakers and citizens, with consideration given to their specific requirements. For instance, one interviewee suggested having different platform versions tailored to different user groups. For example, as mentioned by interviewee 5, including children in the planning and design of playgrounds would require a more playful platform. Another suggestion was making buttons larger for elderly users, as highlighted by interviewee 1.

### (c) Citizen survey

#### (i) Process challenges

In relation to process challenges, the survey focused on understanding how citizens perceive effectiveness in public participation. Transparency, efficiency and responsiveness were identified as the most valued attributes for citizens (20%, 15% and 14%, respectively), followed by trust (13%) and accountability (10%). Conversely, having equity in terms of equal access and obtaining consensus were seen as less important. When given the opportunity to provide input in an open box, respondents provided additional insights on what makes participation effective: the ease of obtaining information, the ease of accessing participatory activities or digital platforms and the use of multiple languages. Interestingly, when asked about their participation preference, most respondents prefer to participate in urban planning via digital technologies (43%) or in a hybrid setting (37%); only 10% prefer in-person activities, while the remaining 10% prefer not to participate at all. Concerning the combination of digital tools with physical interactions, some respondents highlighted the importance of public hearings and workshops to allow citizens to provide input in a more personal manner. As one respondent stated, ‘Physical meetings are much more personal and would underline the seriousness and valuation of an individual’s opinion’. The context of planning decisions was also highlighted, emphasizing the importance of understanding how past decisions affect the present. Another interesting insight is the interest of citizens in monitoring and following up on participatory activities; as stated, they would like to receive updates on the topics they are engaged in.

#### (ii) Platform requirements

Citizens were asked about what would encourage or discourage their involvement in a digital participation platform. Regarding what encourages them, ease of use emerged as the most prominent factor (27%). Following closely was the possibility of finding information regarding the planning decisions being made (19%), which is related to the third most selected factor: the importance of the platform covering topics relevant to their daily lives, concerns and needs (17%). In addition, 12% of respondents selected the power to influence planning decisions as a motivating factor. Notably, gamification elements and monetary rewards were among the least nominated motivators, each accounting for 4% of responses. Respondents also highlighted the importance of knowing ‘who is who’ as a motivating factor: who else is involved and who is responsible for taking their input into account.

By contrast, the most discouraging factors were time availability (23%), the perception that their input would not be taken seriously by authorities (19%) and the lack of interest from citizens themselves (18%). It is interesting to note that language and technology barriers were not ranked as prominent factors individually but together account for 28% of the responses: language barriers (12%), access to digital technologies (10%) and digital literacy (6%). Other factors indicated by respondents in the open box include issues with the platform design and interface, inappropriate or irrelevant content, and lack of accountability and continuity. Concerning specific requirements of the platform design, privacy (19%) was selected as the most essential, followed by security, accessibility and user-friendliness (17% each). Transparency was also distinctive among the responses, with 13%. It is worth noticing that developing the platform as open source and modular are requirements that respondents did not consider essential for a public participation platform (6% and 2%, respectively).

#### (iii) Platform features

Citizens were also asked about their views on the essential features of a digital public participation platform. Voting emerged as the most favoured feature to provide input (23%), followed by the possibility of ranking options (16%) and the possibility of interacting on a map (16%). The inclusion of open-ended questions was also deemed important (15%). Additionally, 10% of respondents highlighted the importance of uploading pictures related to specific issues or areas, 8% would like to interact in a three-dimensional environment and 6% find storytelling important. Uploading videos or using audio recordings were the least preferred options (5% and 1%, respectively). Furthermore, respondents offered additional input, such as the opportunity to submit their own suggestions (open feedback). Interestingly, three respondents reiterated the need to tailor the features to specific use cases.

## 4. Synthesis

The findings previously presented indicate that the urban actor groups have somewhat similar expectations and needs regarding process challenges, prioritizing factors such as trustworthiness and transparency, but diverging views regarding other aspects, such as some platform features. Furthermore, it is evident that adopting a citizen-centric approach to public participation is essential, entailing the customization of platform design—including features, content and language—to accommodate diverse user cases and needs. To translate the results into concrete outputs, we created two sets of guidelines (table 1): (i) guidelines for effective public participation and (ii) guidelines for designing urban participation platforms.

**Table 1.** Multi-actor guidelines for effective public participation and for designing digital participation platforms. Numbers are used for identification only and do not imply any order of importance.

guidelines for effective public participation	
	1.1. assess the effectiveness of public participation from a multi-dimensional perspective (both process and outcome);
	1.2. ensure that processes are suitable for the context and stakeholders and tools/methods are tailored to use cases and users;
	1.3. build trust by ensuring transparency in the process, with attention to expectation management;
	1.4. ensure inclusiveness, keeping in mind that being involved is not the same as feeling engaged or having actual influence in the decision-making process;
	1.5. combine top-down and bottom-up approaches;
	1.6. be open to and invest in new partnerships to straighten local networks;
	1.7. build a knowledge basis to establish a level playing field;
	1.8. consider having a moderator in the process;
	1.9. combine digital and physical participation;
	1.10. beware of reciprocity and rewards;
	1.11. make sure resources are in place within institutions and the public;
	1.12. connect public participation to the everyday life of citizens.

guidelines for designing digital participation platforms	
	2.1. take a user-centric approach;
	2.2. develop user-friendly interfaces to ensure intuitive navigation and an easy-to-use experience;
	2.3. provide opportunities for stakeholder exchange and dialogue;
	2.4. contextualize the platform content to provide a sense of community;
	2.5. make sure the content is relevant, up to date and tailored to the use case and related users;
	2.6. use appropriate and contextual language/communication (spoken language and formality level);
	2.7. beware of the trade-offs between transparency and open data and privacy and security;
	2.8. combine qualitative and quantitative data for more meaningful content;
	2.9. invest in attractive visuals;
	2.10. consider both the quality and quantity of engagement;
	2.11. create a sense of trust among platform users;
	2.12. tailor the platform features (table 2) to the use case and related users.

To create the guidelines, we followed an aggregative synthesis process in which the workshop and interview codes and the survey results were combined to provide a comprehensive summary of what actors find important for effective public participation and for designing digital participation platforms. Some guidelines are aggregated based on common concerns, and others are based on tensions between actors, with the way they are phrased indicating these differences. For example, guidelines 1.2 and 1.4 cover common concerns of all actor groups and their importance is highlighted by the imperative of ‘ensure’, while guideline 1.10 highlights the concerns around reciprocity and rewards, with the imperative ‘beware’, asking for caution. We emphasize that these guidelines are a checklist but as pointers to be discussed and contextualized in each participatory situation. In addition, the platform features identified in this study are listed in table 2. Note that the results of the citizen survey represent the most popular features chosen by the survey respondents (complete results can be found in the electronic supplementary material).

**Table 2.** Summary of platform features (workshops (W), interviews (I) and citizen survey (C)). The results of the citizen survey represent the most popular features chosen by the survey respondents (complete results can be found in the electronic supplementary material).

platform features			
open/closed questions	W		C
voting			C
ranking			C
open feedback	W		C
maps	W	I	C
storytelling	W	I	
gamification	W	I	
social Media		I	
three-dimensional models and visualizations		I	
LIDAR scanners		I	
audio recordings		I	
wearable technology		I	
user verification	W	I	
QR codes	W	I	
pictures upload			C

Finally, based on existing conceptualizations of public participation [38–40], the multi-actor insights presented previously, and the survey of existing platforms, we identified five characteristics that help differentiate types of urban platforms: objective, maintenance/moderation (related to the *facilitators* in [40]), main users (related to the *participants* in [40]), level of participation [28,29] and the content (including type of data, frequency of updates and language or tone). Taking these characteristics into account, we propose a typology with six types of urban platforms, synthesized in table 3 and described next.

**Table 3.** A typology of urban participation platforms.

	city dashboard	monitoring platform	spatial survey tool	neighbourhood identity platform	community action platform	neighbourhood forum
objective	city overview	monitoring of projects or programmes	spatial data collection	community building	self-organization	social media/mutual aid
maintenance/moderation (facilitators)	municipality	project/programme leaders	municipality or research institutions	neighbourhood managers or community organizations	local community, individual citizens	municipality, individual citizens
main users (participants)	local governance actors	local governance actors	individual citizens	local community, local governance actors	local community, individual citizens	individual citizens
level of participation	information	information	consultation	partnership/information	delegation/citizen control	delegation/citizen control
content/data	quantitative	quantitative and qualitative	quantitative and qualitative	qualitative	qualitative	qualitative
content/updates	yearly	monthly	monthly	monthly	monthly/ weekly	weekly/daily
content/language	formal	formal	formal/informal	informal	informal	informal
example	Onderzoek010 Rotterdam^a^	Resilient Rotterdam website^b^	Consul Democracy^c^, FixMyStreet^d^, DIPAS platform^e^	Bospolder-Tussendijken website^f^	Mathenesse aan de Maas website^g^ and Instagram account^h^	Neighbourhood WhatsApp groups, Front Porch Forum^i^, FragNebenan^j^

^a^ onderzoek010.nl

^b^ resilientrotterdam.nl

^c^ consuldemocracy.org

^d^ fixmystreet.com

^e^ dipas.org

^f^ bospoldertussendijken.nl

^g^ mathenesseaandemaas.nl

^h^ instagram.com/mathenesseaandemaas

^i^ frontporchforum.com

^j^ fragnebenan.com

1. The *city dashboard* provides a centralized overview of various aspects of urban life and governance, serving as a resource for local government actors and stakeholders involved in urban planning and policy-making processes. It offers a detailed city overview, presenting key information and data on different aspects of city functioning, such as demographics, infrastructure and services. These data do not require frequent updates (annual or less frequent). The platform also provides comparative analyses between neighbourhoods or districts to inform decisions on resource distribution within the city. The platform utilizes quantitative indicators to present data in a structured and measurable format, facilitating informed decision-making and analysis. Since the platform is integrated into municipal systems and governance structures, it adopts a formal language and appearance. This type emerged from the need to share information vertically across local urban governance actors identified in the workshops. An example is the Onderzoek010 Rotterdam platform.
2. The *monitoring platform* is designed to track and oversee projects or programmes within a specific geographic area. It focuses on monitoring the progress of projects or initiatives within a larger programme, offering quantitative indicators to measure key metrics and qualitative information for a deeper understanding of outcomes (i.e. reports and newsletters). The platform’s moderation lies with project or programme leaders, and the main users are local governance actors, as they wish to accompany the progress of local projects and programmes. The frequency of platform updates depends on the project/programme duration, with shorter projects requiring more frequent updates. As the platform serves stakeholders closer to communities and citizens, the tone and interface can be less formal than the city dashboard. This type also responds to the need to share information vertically across local urban governance actors identified in the workshops. An example is the website of Resilient Rotterdam.
3. The *(spatial) survey tool* aims to gather specific information from stakeholders within a defined area. This platform requires input from two actor types: the survey designer (researchers or governance actors) and the survey respondent (any other stakeholder, including citizens). The survey characteristics (questions, duration, frequency, etc.) depend on the survey purposes (research or public interests) and the targeted respondent group (spoken language, level of formality, technical versus local and experiential knowledge, etc.). Similarly, the platform moderation lies either with research institutions or with the municipality. Partnerships between the two are also common in this case. Through this collaborative approach, citizens often become the main focus of the platform, contributing valuable insights and local knowledge to inform decision-making processes. This type emerged from both the academics and governance actors, who had a strong interest in understanding the lived experiences and preferences of people in the city through data collection. A typical example here is the use of digital platforms for participatory budgeting, in which citizens can propose projects and/or vote on selected projects (e.g. Consul Democracy). Other examples are FixMyStreet and the DIPAS platform.
4. The *neighbourhood identity platform* aims to strengthen local networks and foster community building within neighbourhoods. Updated on a more frequent basis, it prioritizes qualitative data to portray the unique identity and needs of each neighbourhood. Managed either by local governance actors at the neighbourhood level or by local community organizations, the platform serves as a space for local community members and governance actors to collaboratively identify and celebrate the distinct characteristics and strengths of their neighbourhood. With an informal interface, the platform also empowers individual citizens to actively participate in shaping the identity and future direction of their neighbourhood. This type aims to support the creation of new partnerships and leverage the untapped potential of existing local networks and resources while avoiding neighbourhood stigmatization by co-creating the portrait of the neighbourhood. An example is the district Bospolder-Tussendijken in Rotterdam, which maintains a website with personal stories from residents, existing initiatives in the district, and calls to fund new initiatives and projects.
5. The *community action platform* seeks to empower local communities to collaborate and take the initiative in addressing their own needs and interests. It provides a space for organized groups and individual citizens to share ideas, organize events and mobilize resources to improve their community, fostering a sense of ownership and empowerment within the local community. Given the fast dynamic of communities, the platform operates on a frequent basis and is managed by local community organizations or individuals (e.g. volunteers). The interface and language are both informal. This type also aims to transfer control to the community and individual citizens. An example is the community organization Mathenesse aan de Maas, active in Oud-Mathenesse, a neighbourhood in Rotterdam. The organization maintains a website and an Instagram account, which serve as a platform for event organizations and calls for action within the neighbourhood.
6. The *neighbourhood forum* is a platform type leveraging social media to facilitate exchange and engagement among individual citizens. With a dynamic virtual space, the platform aims to foster community cohesion and mutual aid, promote local initiatives and share resources within the neighbourhood. Here, residents actively participate by contributing to discussions and sharing their opinions, concerns and ideas about neighbourhood matters in real time. While adopting an informal interface, oversight may be provided by the municipality to ensure a safe environment and safeguard privacy. By contrast, ownership and moderation by citizens can be more empowering as both improve transparency and keep the data under citizen control. Examples are the Front Porch Forum and FragNebenan platforms. Another example is the creation of neighbourhood groups in messaging apps (such as WhatsApp).

## 5. Discussion

The paper examines the contentious issue of public participation in urban planning and policy in the context of the increasing digitalization of participatory practices. It explores the complexity of public engagement, the definition of effective participation and the use of digital platforms for engagement. Drawing from workshops with local governance actors, interviews with academics and a citizen survey, we identify three emerging themes: *challenges* of public participation within urban governance, perspectives on the *requirements* of digital participation platforms, encompassing aspects from privacy to platform content, and platform design *features* that address challenges and/or achieve platform requirements, discussed next.

Concerning challenges in public participation processes, the three urban actor groups agreed on various aspects. Across workshops, interviews and the citizen survey, the importance of clear communication, trust and transparency among stakeholders involved in the planning process was emphasized, corroborating previous research [16,30,64,65]. Notably, citizens and academics have stressed the importance of accountability, efficiency, responsiveness and continuity (e.g. through monitoring and updates). However, there seems to be a divergence regarding the intents of public institutions. While governance actors ensure that the municipality is interested in citizen input and values citizen knowledge, academic actors expressed concerns about the dominant top-down approach to urban governance and the associated resistance to involving citizens meaningfully in planning and policy. This tension may be related to the technocratic approach to participation resulting from the institutionalization of these practices [15,16] and potentially indicates the importance of (re)defining the very meaning of participation, bringing back the binary debate about whether participation is a democratic tool that meaningfully involves citizens in decisions or a problem-solving tool used within a technocratic context.

In addition, all groups stressed the importance of understanding the context of planning decisions and the need to tailor participation methods and tools to different user groups and contexts, including combining digital and in-person activities [66]. Regarding the use of monetary rewards, both governance and academic actors expressed concerns about their impact on citizen opinions and manipulation. Citizens, by contrast, do not necessarily value monetary rewards. Non-monetary incentives and societal motivations were highlighted as alternatives to monetary rewards, which aligns with the literature [21,67]. Other important insights from this study include the role of new partnerships in strengthening local networks to (indirectly) improve stakeholder engagement, the need to make participation relevant to the everyday lives of citizens (including the platform content), the importance of communication in ensuring engagement (formal versus informal tone, risks of stigmatization) and the need for a moderator both for the participatory process and for digital platforms.

In relation to platform requirements, the three actor groups agreed on the importance of user-centric designs and easy-to-use platforms for effective public participation and highlighted the importance of intuitive layouts and visually appealing interfaces to enhance user engagement. While the need for user-centric approaches is widely recognized in the fields of service design and human–computer interaction [68–70], it is often overlooked in the planning literature. There was also agreement on the importance of ensuring privacy and security in digital participation platforms, including citizens. Trade-offs between privacy and transparency were explicitly discussed by academic actors, which may reflect the recent attention to the topic due to recent updates in GDPR and similar regulations and debates around the use of artificial intelligence. Beyond the trade-offs between open source and proprietary systems, important aspects regarding platform governance were not mentioned, such as the tensions between private and public parties as digital platforms continue to roll out in cities and new power dynamics and forms of colonialism and dispossession emerging with the use of urban platforms and digital technologies [28,34–37,71].

Finally, a total of 15 different platform features have been identified in this study. There was a general agreement concerning the use of maps and the need for different options to provide input. Citizens indicated their preference to provide input through voting, ranking and open feedback. However, perspectives varied on the necessity of features like audio recordings or wearable technologies, with some actors considering them valuable additions while others were more sceptical about their effectiveness and privacy implications. In line with previous research, our study corroborates the potential of social media, three-dimensional models, gamification and mapping features [6,7,22,25,66,72–81].

### (a) Study limitations

By considering the perspectives of different urban actor groups, this research provides concrete insights that support public participation, particularly in the context of increasing incorporation of digital technologies in urban governance. However, the data used in this study entail limitations. The first concerns the context, as the local governance actors and most interviewees in this study work in the Dutch context. The citizen survey sample is more diverse, as it includes the perspectives of residents from other cities, but still, mostly European cities. The second limitation is the study scope. In this work, we focused on governance, academic and citizen perspectives and had to leave out other important actor groups, such as local businesses, non-governmental organizations and activist organizations. These two limitations may explain the absence of or lesser emphasis on other typical topics related to public participation in cities, such as informal and insurgent participatory practices or power struggles over land ownership.

Finally, we highlight that the guidelines and the typology presented in this study reflect the data collected in this study; as such, they are proposed as complementary recommendations and additional types to existing guideline sets and typologies, respectively. It is worth noting that the typology emerging from the empirical data challenges the definition of digital platforms assumed at the beginning of the study. Types 5 and 6 respond to the need for self-organization and mutual aid to foster community cohesion and empowerment. These types connect citizens to each other and to other local actors, strengthening social networks and local support structures, facilitating mutual connections and supporting neighbourhood initiatives. The collective power of a strong community amplifies its potential to impact decisions, influencing their relations and ability to negotiate with public institutions. These types, however, fall out of the definition used earlier [62], which does not consider social media and other apps and websites as digital participatory platforms. A systematic review of guidelines and typologies is out of the scope of this paper but presents a promising direction for future work to consolidate existing knowledge, solve conceptual tensions and provide concrete directions for research and practice.

## 6. Conclusion

Over the past decades, public participation has become an integral component of urban planning and policy in many Western countries. Yet, despite its widespread adoption and the advancements in participatory digital technologies, the effectiveness of public participation remains a subject of ongoing debate. In contrast to, but building upon, theoretical accounts of public participation, this study explored the perceptions of urban actors concerning public participation in the context of increasing digitalization. Adding to the empirical literature on the topic, we sought a multi-actor perspective, focusing on local governance actors, academics and citizens.

Through a combination of workshops, interviews and a citizen survey, this study thus offers a comprehensive understanding of the challenges faced by various urban actors in participatory processes and citizen engagement. The results showed that urban actors have similar expectations of public participation processes and value similar factors, such as clear communication, trust and transparency. Additionally, concerning digital platforms, there was agreement on the need for a user-centric approach that tailors the platform (its design, features and content) to different use cases and user needs. However, diverging views were also observed, markedly concerning the intentions of public institutions, incentives and motivations to participate and some platform features. While these results indicate that there is a common ground on which actors can build to improve participation, critical questions about intentions and motivations on both proponents’ and participants’ sides remain open. Solving these tensions may require engaging actors in redefining public participation, not only processes and digital tools/platforms but the very meaning of participation, which brings us back to the binary debate of whether participation should be a democratic tool that appeals to intrinsic and societal motivations or citizens (and other actors) to participation or a problem-solving tool that may appeal to their personal, ‘selfish’ motivations, or whether it should and could be both.

Without ignoring these critical questions, this paper also offers practical guidelines for effective public participation, which can be helpful in the design and planning of participatory activities, and recommendations for designing digital participation platforms, including a list of promising platform features. These guidelines are not proposed as a checklist but as pointers to be contextualized in each participatory situation. In addition, we propose a typology of digital participation platforms that acknowledges the diverse needs and preferences of different urban actors, offering a multi-actor framework for the evaluation and refinement of such platforms. Our typology is, however, partially at odds with the existing literature on participatory platforms, and therefore we suggest a systematic review of typologies as a promising research direction to shed light on these conceptual tensions.

## Data Availability

The survey data have been made available via the 4TU Repository: [82]. (Raw) data collected during the workshops and the interview transcripts will not be made available to preserve the privacy of participants. Supplementary material is available online [83].

## Footnotes

^1^
In this paper, we understand governance as described by Edgar Pieterse in his *Working Notes on a Relational Model of Urban Politics*, which is based on Patsey Healy’s work: ‘Governance denotes the relationality of power as it flows through networks between the state and institutional actors in the market and civil society’ [61]. However, ‘governance is not a homogenous agent, but a morass of complex networks and arenas within which power dynamics are expressed and deployed’ [1].
